# The Effects of GLP-1 Agonists on Musculoskeletal Health and Orthopedic Care

**DOI:** 10.1007/s12178-025-09978-3

**Published:** 2025-05-15

**Authors:** Andrew Gatto, Kevin Liu, Nesa Milan, Stephanie Wong

**Affiliations:** Department of Orthopaedic Surgery, University of California San Francisco, San Francisco, CA USA

**Keywords:** GLP-1 Agonists, Orthopedic Surgery, Skeletal Muscle, Joint, Bone, Outcomes

## Abstract

**Purpose of Review:**

The global rise in obesity and type 2 diabetes mellitus (T2DM) presents significant challenges in musculoskeletal care, contributing to increased perioperative complications, impaired bone health, and compromised muscle function. Glucagon-like peptide-1 receptor agonists (GLP-1RAs), initially developed for glycemic control in T2DM, have demonstrated substantial benefits in weight reduction and metabolic regulation. The purpose of this review is to understand the musculoskeletal biologic and clinical implications of GLP-1RAs.

**Recent Findings:**

Evidence suggests that GLP-1RAs may impact musculoskeletal health through anti-inflammatory effects, bone metabolism modulation, and alterations in muscle composition. GLP-1RAs may promote osteoblastogenesis while dampening osteoclast activity to maintain bone mineral density. The result on fracture risk is unclear. Additionally, while GLP-1RAs cause lean mass loss, GLP-1RAs appear to preserve skeletal muscle, reduce fatty infiltration, and enhance fiber formation and function. Further, GLP-1Rs are present in synovial tissue and cartilage, demonstrating downregulation of inflammatory molecules and chondrocyte apoptotic pathways, though clinical studies show variable effects in the setting of osteoarthritis. Overall, the heterogeneity in findings underscores the need for further research to delineate the long-term musculoskeletal effects of GLP-1RAs.

**Summary:**

Understanding the musculoskeletal impact of GLP-1RAs is critical for optimizing their integration into orthopedic practice. This review explores the orthopedic implications of GLP-1RAs, highlighting their biologic mechanisms and clinical effects on obesity-related joint inflammation and arthropathy, bone mineral density and fracture risk, and skeletal muscle preservation.

## Introduction

The global surge in obesity and type 2 diabetes mellitus (T2DM) presents significant challenges in orthopedic care, directly impacting musculoskeletal health and surgical outcomes. These metabolic disorders are associated with increased rates of perioperative complications, including infections, wound dehiscence, and prolonged recovery following orthopedic procedures, underscoring the urgent need to address these systemic conditions within the context of orthopedic care [1, 2]. ^,^ [3, 4]

Obesity and diabetes have a well-established risk for postoperative infections. Andrade et al. demonstrate that obese patients have a 4.7 times higher risk for infections compared to their normal-weight counterparts, with a 6-fold increase in morbidly obese individuals [5]. Terada et al. [6], observed a strong association between diabetes and increased postoperative infection rates, and Mauro et al. [7] similarly demonstrated that the coexistence of diabetes and obesity significantly raises the likelihood of periprosthetic joint infections following total joint arthroplasty. Additionally, the negative impact of diabetes on bone integrity, evidenced by increased cortical porosity and impaired fracture healing, presents additional challenges in the orthopedic management of these patients [8]. 

Recognizing the detrimental effects of metabolic dysregulation, glucagon-like peptide-1 receptor agonists (GLP-1RAs), such as semaglutide and liraglutide, have emerged as promising therapeutic agents. Though initially developed for T2DM management, large trials such as Semaglutide Unabated Sustainability in Treatment of Type 2 Diabetes (SUSTAIN) [9] and Semaglutide Treatment Effect in People with Obesity (STEP) [10] have consistently shown substantial decreases in body weight and optimization of metabolic parameters in patients with T2DM and obesity [11, 12]. 

Beyond their metabolic benefits, emerging evidence suggests that GLP-1RAs may offer additional advantages for musculoskeletal health. Preclinical and clinical studies indicate that these agents possess anti-inflammatory properties and may positively influence bone metabolism, muscle composition, and joint homeostasis through preserving bone mineral density, reduce muscle fatty infiltration, and alleviate joint inflammation, respectively [1, 13]. These effects suggest a possible therapeutic role in orthopedic care.

The potential of GLP-1 receptor agonists to improve glycemic control, promote weight reduction, and enhance musculoskeletal health presents a promising avenue for reducing the burden of obesity and T2DM in orthopedic practice. The purpose of this review is to understand the musculoskeletal biologic and clinical implications of GLP-1RAs.

### GLP-1 Receptor Agonists

GLP-1 plays an essential role as an incretin hormone, significantly influencing glucose metabolism, appetite regulation, and systemic health. Secreted from intestinal L-cells in response to food ingestion, GLP-1 enhances glucose-dependent insulin secretion while suppressing glucagon release, thereby promoting effective glycemic control [14–16]. In addition, GLP-1 receptor activation increases satiety, slows gastric emptying, and improves caloric impulse control, mechanisms critical for weight management [17, 18]. Semaglutide and Liraglutide, two widely used GLP-1RA FDA-approved for weight loss (Table 1), have demonstrated effectiveness by reducing energy intake as a result of both direct and indirect effects on central appetite-regulating pathways [19, 20]. 

The GLP-1 receptors (GLP-1R) are distributed across various tissues, including those of the musculoskeletal system [21–23]. This binding not only modulates insulin and glucagon dynamics but also triggers anti-inflammatory responses that help mitigate chronic low-grade inflammation, a hallmark of obesity-related comorbidities [24].

**Table 1:** Available GLP-1 agonists

Duration	Drug Name	Trade Name	Dosing (mg)^	Molecular Structure
Long	Semaglutide	Ozempic	0.5-2.0 weekly	GLP-1 analog with fatty acid side chain for albumin binding
		Rybelsus	7.0–14.0 daily 4.0–9.0 daily	
		Wegovy*	1.7–2.4 weekly	
	Dulaglutide	Trulicity	0.75–4.5 weekly	GLP-1 analog fused to Fc fragment of IgG4
	Liraglutide	Victoza	1.2–1.8 daily	GLP-1 analog with fatty acid for albumin binding
		Saxenda*	0.6-3.0 daily	
	Tirzepatide	Mounjaro	5.0–15.0 weekly	**Dual GLP-1/Gastric inhibitory polypeptide(GIP) receptor agonist**, fatty acid modification for albumin binding
		Zepbound*		
	Exenatide	Bydureon	1.0 weekly	**Exendin-4-based** peptide (53% homology to human GLP-1)
Short		Byetta	5.0–10.0 mcg twice daily	
	Lixsenatide	Lyxumia	20 mcg daily	**Exendin-4-based** with C-terminal modifications

^Dosing listed as maintenance dosing, after dose escalation to avoid adverse effects

*FDA approved for weight loss

Dosing guides retrieved from UpToDate Lexidrug (Wolters Kluwer)

### Relevance To Orthopedic Care

Excess body weight increases mechanical stress on joints, contributing to osteoarthritis, impaired tissue regeneration, and an elevated risk of fractures [25]. By promoting weight loss and improving metabolic profiles, GLP-1RAs enhance overall musculoskeletal function with alleviated joint stress, improved healing potential, and greater tissue quality [26]. Their anti-inflammatory properties could also reduce systemic inflammation, thereby potentially improving recovery after orthopedic surgeries and attenuating pain associated with inflammatory joint diseases [2, 27–29]. 

Given the promising findings regarding GLP-1 agonists in managing metabolic conditions and their beneficial repercussions on musculoskeletal and orthopedic health, there is potential for integrating these agents in orthopedic care protocols [2, 30]. This integration could be beneficial in post-surgical rehabilitation or in managing chronic musculoskeletal pain through their anti-inflammatory and metabolic effects [1].

## The Biologic Effects of GLP-1 Agonists on Musculoskeletal Tissue

### Bone Structure and Metabolism

#### Calorie Restricted Weight Loss and Bone Health

Calorie-restriction weight-loss, the mechanism induced by GLP-1-RAs, is associated with reductions in bone mineral density (BMD) with increased bone turnover [31]. Such findings may be a physiologic response, as explained by the mechanostat theory which posits that reduced skeletal strain leads to adaptive bone reabsorption. As such, it is usual to expect diminished BMD with weight loss. In fact, a 10% reduction in body weight has been associated with 2.2% decrease in lumbar and total hip BMD [31]. Further, diet induced weight loss was associated with significant decreases in BMD at the total hip (− 2.2% ± 3.1%) and intertrochanter (− 2.1% ± 3.4%) [32]. These findings highlight a clinically relevant pattern of bone loss, particularly evident with rapid, calorie-restricted weight loss, such as that seen with GLP-1-RAs [33]. 

#### GLP-1RAs Modulate Bone Metabolism

Emerging evidence indicates that GLP-1RAs may help mitigate bone loss associated with weight loss by acting at the cellular level. GLP-1Rs have been identified on osteoblasts, osteoclasts, and bone marrow stromal cells (BMSCs) [34–36]. In preclinical studies, increased GLP-1 receptor expression has been observed during the osteogenic differentiation of adipose-derived stem cells [21]. Liraglutide has been shown to enhance human osteoblastogenesis in vitro, as evidenced by increased mRNA expression of the osteoblast-specific transcription factor, Runx2, along with osteogenic markers alkaline phosphatase (ALP) and collagen type I alpha 1 (Col1A1), suggesting a potential role for GLP-1 in regulating osteoblast differentiation [37]. 

The effects on osteoclast activity are complex. In ovariectomized mice (a validated model for osteoporosis), extendin-4 treatment decreased the expression of sclerostin in osteocytes, a key suppressants of bone formation [34, 38]. This affect, however, was not demonstrated with liraglutide, highlighting possible differences in affect among GLP-1 analogs. Additionally, Pereira et al., found that GLP-1RA increased the number of osteoclast while modestly reducing their resorption area, a paradox underscoring further unknown mechanisms [34]. Further, GLP-1RA act on thyroid C-cells, increasing calcitonin levels, thereby promoting osteoprotegin expression and inhibiting receptor activator of nuclear factor-κB ligand (RANKL), effectively reducing osteoclast-mediated bone resorption [34, 39, 40]. 

#### Bone Structure Response

The cellular effect of GLP-1RA is demonstrated clinically in BMD. GLP-1RAs modulate serum biomarkers of bone formation (e.x., b-ALP, OC, and P1NP) and bone resorption (e.x., CTX) (Table 2). Iepsen et al. induced weight loss in obese women prior to initiating low-dose liraglutide (1.2 mg/day) and found that the weight-loss–associated decreases in BMD were abolished with GLP-1RA treatment while weight loss was maintained [3]. A systematic review of 25 studies found that GLP-1RA treatment over 6 months resulted in significantly greater improvement in BMD at the lumbar spine and femoral neck, particularly in patients with T2DM over 55 years old [41]. However, Hansen et al. and Jensen et al. found an opposite effect with non-T2DM taking GLP-1RAs for weight loss [33, 42]. Another analysis on treatment duration demonstrated an increase in lumbar spine BMD with treatment ≤ 26 weeks and ≥ 44 weeks, suggesting influence by treatment duration [43]. 

GLP-1RA may affect cortical and trabecular bone. Trabecular bone has demonstrated improved mass, connectivity, and structure, with preserved cortical bone in mice treated with of GLP-1RA [34]. In GLP-1R knockout mice, cortical bone mass and strength were significantly reduced [44]. Liraglutide has demonstrated greater preservation of cortical bone mass during weight loss, compared to controls [33, 42]. However, in a study demonstrating reduced BMD, cortical thickness and porosity were negatively affected in human tibia, though not in the radius; notably, trabecular bone and bone strength were unchanged [33]. Discrepancies in results may be attributed to skeletal adaptions in response to mechanical loading.

Overall, the influence of GLP-1RAs on bone metabolism appears multifaceted. While GLP-1RAs may exert a protective effect, their impact may depend on patient age, metabolic status, treatment dose and duration, and degree of weight reduction. Exercise repeatedly demonstrates similar weight loss with reliable preservation of bone [42, 45, 46]. Further high-quality, long term studies are warranted to delineate the specifical skeletal effects of GLP-1RAs.

**Table 2:** Influence of GLP-1RAs on bone metabolism biomarkers

Measures	Bone Turnover Markers	Study Results
Bone Resorption Markers	**C-terminal telopeptide of type I collagen (CTX)**	**^MD: −0.36 (95% CI − 0.70**,** − 0.03)**[46] **^MD :– 0.34 (95% CI– 0.54 to– 0.14)** [36] **^MD: +0.04 (95% CI 0.01–0.07 µg/L)**[112] **MD: +0.06 (95% CI 0.009 to 0.1)**[3]
	N-terminal telopeptide of type I collagen (NTX)	PP: 0.0% (95% CI − 84.2% − 82.6%)[113]
Collagen maturation	**procollagen-1 N-terminal peptide (P1NP)**	^MD: −0.36 (95% CI − 4.48–3.76 µg)[112] MD: −0.1 (95% CI − 3.2–3.0)[3] **^MD : +0.45 (95% CI 0.01–0.89)**[46] **^MD = + 0.33 (95% CI 0.07–0.59)** [44] ETD : 3.84 (95% CI −5.6–13.3)[36]
Bone Mineralization	osteocalcin (OCN)	MD: −1.4 (95% CI − 2.5 - −0.2)[3] **^MD: +2.04 (95% CI 0.99–3.08)**[46] **MD = + 1.46 (95% CI 1.10 to 1.83)** [44]
	bone-specific alkaline phosphatase (B-ALP)	^MD: −1.24 (95% CI − 3.02–0.53 µg/L)[112] MD: −0.78 (95% CI − 0.29–1.8)[3] **^MD = + 0.91 ug/L (95% CI 0.19 to 1.63)** [44] **^MD: +0.76 (95% CI 0.29–1.24)**[46] PP : 7.42% (− 29.6 − 42.8%)[113]
Bone Remodeling/Bone Mass	NFkB ligand (RANK-L)	^MD: −0.08 (95% CI − 1.80–1.65)[112] PP : 45.07% (95% CI − 53.3% − 930.9%)[113]
	osteoprotegerin (OPG)	PP: −31.9% (95% CI − 81.6% − 62.7%)[113]

MD: Mean difference. ETD: Estimated Treatment Difference.; PP: pre- and post-treatment % change from baseline

^Results from metanalysis

### Skeletal Muscle Structure, Composition, and Function

A growing body of literature demonstrates that GLP-1RAs are associated with significant reductions in lean body mass. Up to 40% of overall weight loss with semaglutide, and up to 25% with tirzepatide, is attributed to lean body mass, which raises concerns that the drug therapy may hold a negative effect upon skeletal muscle mass and function [10, 47]. Because individuals with obesity tend to have greater muscle mass, they are expected to lose a significant amount of muscle during weight loss. It is important to consider whether this muscle loss is an adaptive or maladaptive response. Understanding the impact of GLP-1RAs on muscle quality and function is critical to understanding the fundamental metabolic role of GLP-1RAs.

#### Skeletal Muscle Mass

Research on the effects of GLP-1RAs on skeletal muscle is limited, as much of the existing literature relies on the nonspecific metric of lean body mass, which is defined as skeletal muscle, bone, water, and organ mass. Dual-energy X-ray absorptiometry (DEXA) and bioelectric impedance analysis are methods commonly used to measure lean mass, though it does not differentiate between skeletal muscle and other lean tissue. This context is important to understand when appraising research on the effect of GLP-1 on skeletal muscle.

The skeletal muscle mass lost in GLP-1RA therapy may be proportional to the overall weight loss. Using bioelectric impedance analysis, Ozeki et al. reported that Semaglutide preserved skeletal muscle mass percentage of the extremities (i.e., muscle weight/total weight) despite a decrease in total muscle mass [50]. Uchiyama, et al. found that skeletal muscle index (SMI), a calculated sum of limb lean mass divided by height squared, remained unchanged after Semaglutide treatment [51]. Volpe et al., reported a significant decrease in fat mass index (FMI, whole-body fat/height squared;-3.04 ± 0.43 kg/m^2^) and a modest decrease in SMI (-0.51 ± 0.14 kg/m^2^). Notably SMI stabilized after 3 months, with handgrip strength was unchanged after 6 months of treatment [52]. Overall, GLP-1 receptor agonists (GLP-1RAs) have been associated with reductions in lean mass proportional to total body weight loss, comparable to those observed with other weight loss interventions and glycemic control medications [48, 49]. However, further research is needed to specifically evaluate their effects on skeletal muscle mass.

#### Muscle Quality & Composition

Recent studies of the GLP-1RAs used magnetic resonance imaging (MRI) to evaluate muscle composition quantification and muscle volume z-score, which describes how much an individual’s muscle volume deviates from what is expected for people with the same sex and body size (similar to Osteoporosis z-score) [48, 53–55]. In an evaluation of thigh muscle in non-T2DM, overweight patients by Pandley et al., 69.9% of patients on liraglutide experienced a mean reduction in thigh muscle fatty infiltration of 2.87% (vs. 49.1% of placebo with loss of 0.05%). Meanwhile, change in z-score was similar to placebo [53]. Kakegawa et al. observed that liraglutide lowered fatty infiltration in psoas, paraspinal, and abdominal wall muscles of patients with T2DM [56]. Together, these studies implicate improved muscle composition with appropriate decrease in muscle mass.

#### Cellular and Structural Muscle Adaptations

GLP-1 signaling has a direct effect on muscle quality and performance. In murine skeletal muscle, GLP-1R activation increased glycogen storage, increased glucose uptake, and enhanced mitochondrial biogenesis [57, 58]. Additionally, GLP-1RA promoted type-1 fiber formation with increased size of muscle fiber diameter and sarcoplasmic reticulum indicating an increase in oxidative processes and mitochondrial respiration, one of muscle’s key contributions to metabolic health [57–59]. Further, GLP-1RA demonstrated the ability to reverse poor muscle quality in obese mice models [57, 58]. 

Nevertheless, concerns remain about the long-term impact of sustained GLP-1 exposure on muscle health. Huang et al. found that consistent GLP-1 treatment in mice inhibited myogenic differentiation and glucose uptake through impaired GLUT-4 microtubule translocation and mitochondrial distribution. This same study highlights that sarcopenic individuals demonstrate increased GLP-1 [60]. This indicates that sustained supratherapeutic GLP-1 may actually impact the machinery of myogenesis, and potentially contributes to the development of sarcopenia.

There is a paucity of literature on GLP-1RAs effect on tendons. Abdulmalik et al., observed influence of exendin-4 on tenocyte differentiation in bone marrow-derived human mesenchymal stem cells (hMSCs). Culture media revealed greater proliferation of hMSCs and enhanced gene expression of markers associated with tendon development (e.g., Col-I/III, scleraxis, mohawk gene), seemingly promoting tenogenesis [61]. 

#### Muscle Performance and Function

Muscle performance may be enhanced by GLP-1RAs. Several studies have demonstrated increased secretion of GLP-1 in humans during moderate and intense physical activity [62, 63]. Skeletal muscle microvascular flow is significantly increased in response to GLP-1, leading to an increase in glucose uptake and glycogen storage. Zhang et al. evaluated data from multiple trials and found that GLP-1 receptor agonists significantly improved 6-minute walk test [64]. While difficult to differentiate, enhanced exercise capacity may come from a combined effect of weight loss and direct GLP-1 action on muscle.

Overall, concern that GLP-1RA medications cause large muscle mass loss is not supported by data. Nonetheless, there is heterogeneity in understanding of muscle mass and quality in response to GLP-1RA therapy, underscoring the need for additional research. Readers should be aware of flawed interpretation of data as lean or fat-free mass is not synonymous with skeletal muscle mass [65]. 

### Cartilage and Joint Structure and Function

Immunohistochemical studies have identified GLP-1R in human chondrocytes and synovial membrane sections of knee joints, suggesting that GLP-1-based therapies may have direct effects on joint tissues [22, 23]. Increasing evidence indicates that GLP-1RAs are anti-inflammatory, with a direct effect on macrophage adhesion, and anti-apoptotic effects throughout human tissue [66, 67]. This suggests that GLP-1RAs may modify the inflammatory cascade of osteoarthritis (OA) by modifying the secretion of cytokines and tumor necrosis factor-α (TNF-α), the pro-inflammatory molecules that are produced by macrophages responsible cartilage degradation and synovial inflammation.

#### Modulating Joint Inflammation

Chondrocyte cell signaling is modulated by GLP-1RAs in OA models. Chen et al. observed that activation of GLP-1R was linked to reduced apoptosis and increased cell matrix protection [22]. Notably, GLP-1R expression was diminished in OA cartilage, but treatment with liraglutide restored GLP-1R presence and significantly reduced cartilage degeneration, as evaluated on histology [22, 68]. Que et al. further demonstrated that liraglutide enhanced protein kinase A (PKA) and cAMP-response element binding protein (CREB) signaling in cartilage tissue, reducing expression of TNF-α, IL-6, and IL-1β [68]. 

The activation of GLP-1R has a direct effect on gene expression, downregulating NF-κB phosphorylation and nuclear translocation, thereby reducing the expression of pro-inflammatory cytokines and matrix metalloproteinases (MMPs) [68, 69]. In the monoiodoacetate (MIA) rat model of OA, liraglutide reduced TNF-α-induced upregulation of IL-6 and monocyte chemoattractant protein-1 (MCP-1) by 1.5-fold in a dose-dependent manner.

Anti-apoptotic effects of GLP-1RAs are demonstrated in OA models. Treatment of chondrocytes with liraglutide and dulaglutide decreased MMP-3/13, ADAMTS4/5 expression, which are key drivers of type II collagen and aggrecan degradation, respectively [66, 69]. Furthermore, liraglutide displays an antioxidant effect, reducing TNF-α-induced reactive oxygen species (ROS) and NADPH-oxidase levels, alleviating stress on mitochondrial and endoplasmic reticulum function, and thus reducing chondrocyte apoptosis [22, 69]. These beneficial effects are further augmented by the reduction of advanced glycosylation end products, known stimulators of oxidative stress and inflammation that are elevated in T2DM [66, 70]. 

GLP-1RAs also target synovial inflammation. Liraglutide improved synovitis severity in MIA models, and lixisenatide blunted proinflammatory synovial cell signaling pathways and response [23, 71]. Culture with dulaglutide and human fibroblast-like synoviocytes demonstrated suppressed inflammatory markers such as IL-1β, IL-6, MCP-1, and MMP-3 [72]. These findings highlight GLP-1RA’s promising whole-joint impact.

Overall, agonism of GLP-1R demonstrates an anti-catabolic effect, downregulating oxidative stress, expression of proinflammatory pathways and cytokines, and degradation of articular extracellular matrix. Additionally, the weight-reducing effects of GLP-1 RAs may indirectly benefit joint health by reducing load stress and metabolic inflammation, reinforcing their potential as disease-modifying agents in OA.

### Clinical Implications of GLP-1 Receptor Agonists in Orthopedics

Recent high quality randomized controlled trials (RCTs) have highlighted the clinical benefits of GLP-1RAs, namely improved glycemic control, cardiovascular risk reduction, and weight management [73–76]. Given the interplay between diabetes and obesity on musculoskeletal health, GLP-1 agonists are poised to have a significant impact on orthopedic surgery patients.

#### Impact of GLP-1 Receptor Agonists on Fracture Risk

Current understanding of GLP-1 RA on fracture risk is derived primarily from endocrinology literature. In a meta-analysis of RCTs including T2DM patients treated with either a GLP-1RA or other antidiabetic drugs for greater than 24 weeks, Mabilleau et al. found that GLP-1RAs was not associated with increased fracture risk. However, only 7 of the 28 studies reported fracture outcomes [77]. Similarly, Driessen et al. found no significant change in fracture risk with GLP-1RA use in a retrospective database study [78]. Conversely, a metanalysis by Cheng et al. demonstrated that liraglutide and lixisenatide significantly reduced fracture risk compared to placebo and other anti-diabetic drugs, particularly when used for over 52 weeks [79]. Su et al. also identified a reduced risk of fractures with liraglutide while exenatide was associated with an increased fracture risk [80]. Using a large retrospective database, Venugopal et al. reported that GLP-1RA users with T2DM had lower rates of subtrochanteric, intertrochanteric, and pertrochanteric fractures than those on other diabetic medications [81]. While these findings suggest a potential protective effect of GLP-1RAs, outcomes appear to vary by drug type, duration of use, and fracture site.

#### GLP-1 Receptor Agonist Effect on Joint Disease and Arthroplasty

GLP-1RAs may alter the natural history of arthritis and delay the need for joint replacement through several mechanisms [82, 83]. Using a large-scale multicenter prospective database of 40,000 patients with knee arthritis, Zhu et al., observed reduced cartilage loss, greater weight reduction, and a lower incidence of knee surgery among those taking GLP-1RAs [84]. Similarly, Porto et al. reported GLP-1 RA use decreased the likelihood of conversion to arthroplasty in individuals with pre-existing hip or knee OA and BMI ≥ 30 [85]. 

Symptom relief outcomes remain inconsistent. In a RCT of 409 patients with BMI ≥ 30 and Kellgren-Lawrence grade 2/3 knee arthritis, Bliddal et al. reported that semaglutide resulted in both weight loss and improved knee pain scores [86]. In an RCT by Gudbergsen et al., liraglutide led to significant weight loss without improvements in knee pain [87]. This discrepancy may be attributed to differences in study drug or populations, as the latter included patients with a lower mean BMI and a broader range of radiographic knee arthritis severity.

GLP-1RAs have shown variable effects on outcomes after joint replacement surgery. In a matched analysis of 40,000 total knee arthroplasty (TKA) patients, Magruder et al. reported lower risk of sepsis, periprosthetic joint infections (PJIs), and readmissions in diabetic patients treated with semaglutide versus those without. However, semaglutide was associated with increased risk of myocardial infarction (MI), acute kidney injury (AKI), pneumonia, and hypoglycemia [88]. The same authors analyzed 9,000 matched total hip arthroplasty (THA) patients in a retrospective database study and noted semaglutide users had fewer readmissions within 90 days and PJIs within 2 years [89]. Similarly, in a study of 5,000 TKA patients with BMI ≥ 40, Kim et al. demonstrated GLP-1RA use was associated with lower 90-day PJI, pulmonary embolism (PE), and readmission rates [90]. There were no significant differences in aseptic loosening or prosthetic failure between GLP-1RA users and non-users [90]. 

Regarding total shoulder arthroplasty, Lawand et al. and Elsabbagh et al. found no significant differences in long-term revision rates [91, 92]. However, the first noted higher rates of DVT, MI, pneumonia, and transfusion among GLP-1 RA users within 90 days [91]. 

#### Effects of GLP-1 Receptor Agonists in Spine Surgery

GLP1-RA use has particular relevance in spine surgery. Tao et al. analyzed 596 matched T2DM patients undergoing single or multi-level cervical spine decompression and fusion and did not detect significant differences in short-term complications or 90-day readmissions between semaglutide users and non-users [93]. Regarding lumbar fusion, Khalid et al. found higher 1-year risk of AKI, urinary tract infection, and need for subsequent fusion in patients prescribed semaglutide compared to non-semaglutide users [94]. Seddio et al. reported lower 90-day risk of deep vein thrombosis (DVT) or PE in semaglutide-treated diabetics undergoing posterior lumbar fusion [95]. Finally, Wiener et al. found that in obese diabetic patients undergoing any spinal fusion, GLP-1RA use was associated with reduced infection, readmission, and revision rates compared to no GLP-1RA use [96]. 

#### Effects of GLP-1 Receptor Agonists on Soft Tissue Conditions

There is limited research the interaction of GLP-1RAs and soft tissue pathology. Bergstein et al. reported that diabetic patients on GLP-1RAs had a higher likelihood of developing adhesive capsulitis and requiring operative intervention than those not using GLP-1 RAs [97]. Su et al. found a higher incidence of carpal tunnel syndrome and surgical release in GLP-1 users compared to those on SGLT2 inhibitors [98]. 

Regarding surgical outcomes, Seddio et al. analyzed diabetic patients undergoing arthroscopic rotator cuff repair and found that semaglutide users had a lower risk of surgical site infection, venous thromboembolism (VTE), AKI, and retear at 2 years [99]. Overall, it is unclear how GLP-1RAs may contribute to postoperative complications, highlighting the need for further research.

#### Perioperative Risks

Orthopaedic surgeons should be aware of the potential increased aspiration risk during intubation associated with GLP-1RAs. Case reports have described intraoperative aspiration in patients on semaglutide, likely due to residual gastric contents as a result of delayed gastric emptying [100–102]. Anesthesiology guidelines for perioperative GLP-1RA management remain inconclusive, with current recommendations advising discontinuation between 1 and 4 weeks before surgery [100, 103, 104]. 

With the growing use of GLP-1 RAs for weight loss, concerns about potential malnutrition have emerged. These agents may contribute to nutritional deficits due to behavioral changes in patients undergoing weight loss and their use may be underreported [105]. Surgeons should consider adopting preoperative nutritional surveillance, including albumin and total lymphocyte count, when surgically treating GLP-1RA patients [106, 107]. 

## Conclusion

Overall, GLP-1RAs act on various musculoskeletal tissue, demonstrating a potential benefit to musculoskeletal health and orthopedic outcomes. While robust clinical trials have established their efficacy in glycemic control, weight loss, and cardiovascular protection [2, 108], further research is warranted to elucidate their direct effects on musculoskeletal health and orthopedic pathology. Such investigations will be crucial for safely integrating GLP-1 RAs into comprehensive treatment protocols aimed at optimizing orthopedic care in patients with obesity and/or diabetes.

### Key References

Jensen SBK, Sørensen V, Sandsdal RM, et al. Bone Health After Exercise Alone, GLP-1 Receptor Agonist Treatment, or Combination Treatment: A Secondary Analysis of a Randomized Clinical Trial. *JAMA Netw Open*. 2024;7(6):e2416775. doi:10.1001/jamanetworkopen.2024.16775

This randomized clinical trial directly assesses the effects of liraglutide and exercise on bone health in obese patients without type 2 diabetes, offering key insights relevant to the current treatment paradigm. The study demonstrates that combining liraglutide with exercise leads to superior outcomes.

Pandey A, Patel KV, Segar MW, et al. Effect of liraglutide on thigh muscle fat and muscle composition in adults with overweight or obesity: Results from a randomized clinical trial. *J Cachexia Sarcopenia Muscle*. 2024;15(3):1072–1083. doi:10.1002/jcsm.13445.

This randomized clinical trial presents robust MRI analysis of skeletal muscle composition following Liraglutide treatment, demonstrating reduction in thigh muscle fat and adverse muscle composition compared with placebo.

Mei J, Sun J, Wu J, Zheng X. Liraglutide suppresses TNF-α-induced degradation of extracellular matrix in human chondrocytes: a therapeutic implication in osteoarthritis. *Am J Transl Res*. 2019;11(8):4800–4808.

This preclinical study explores potential mechanisms by which GLP-1RAs may attenuate the inflammatory cascade of osteoarthritis through their effects on human primary chondrocytes, underscoring potential therapeutic applications in OA.

Bliddal H, Bays H, Czernichow S, et al. Once-weekly semaglutide in persons with obesity and knee osteoarthritis. *N Engl J Med*. 2024;391(17):1573–1583.

This randomized controlled trial evaluates the effectiveness of Semaglutide on OA symptoms. In a large, multi-institutional cohort it demonstrates significantly greater reductions in body weight and pain related to knee osteoarthritis than placebo.

Seddio AE, Moran J, Gouzoulis MJ, et al. Lower risk of postoperative complications and rotator cuff retear associated with semaglutide use in patients with type II diabetes mellitus undergoing arthroscopic rotator cuff repair. *Arthroscopy*. 2025;41(2):199–206.

This retrospective database study evaluates rotator cuff repair outcomes in patients with T2DM using semaglutide. The results show decreased odds of adverse events and a lower 2-year rotator cuff retear rate. Overall, the findings support further investigation into the effects of GLP-1RAs on soft tissue healing and surgical outcomes.

## Data Availability

No datasets were generated or analysed during the current study.
